# Supplementary material for: Association between oral microbiome and seven types of cancers in East Asian population: a two-sample Mendelian randomization analysis
Source: Front Mol Biosci. 2023 Nov 21;10:1327893. doi: 10.3389/fmolb.2023.1327893 (PMC10702768; doi:10.3389/fmolb.2023.1327893)
Supplement: Supplementary file 1 [file DataSheet1.ZIP › Supplementary Materials/MR plots for tongue/tongue═╝/Ovarian cancer/pheno.1622_to_ovarian cancer_forest.pdf]

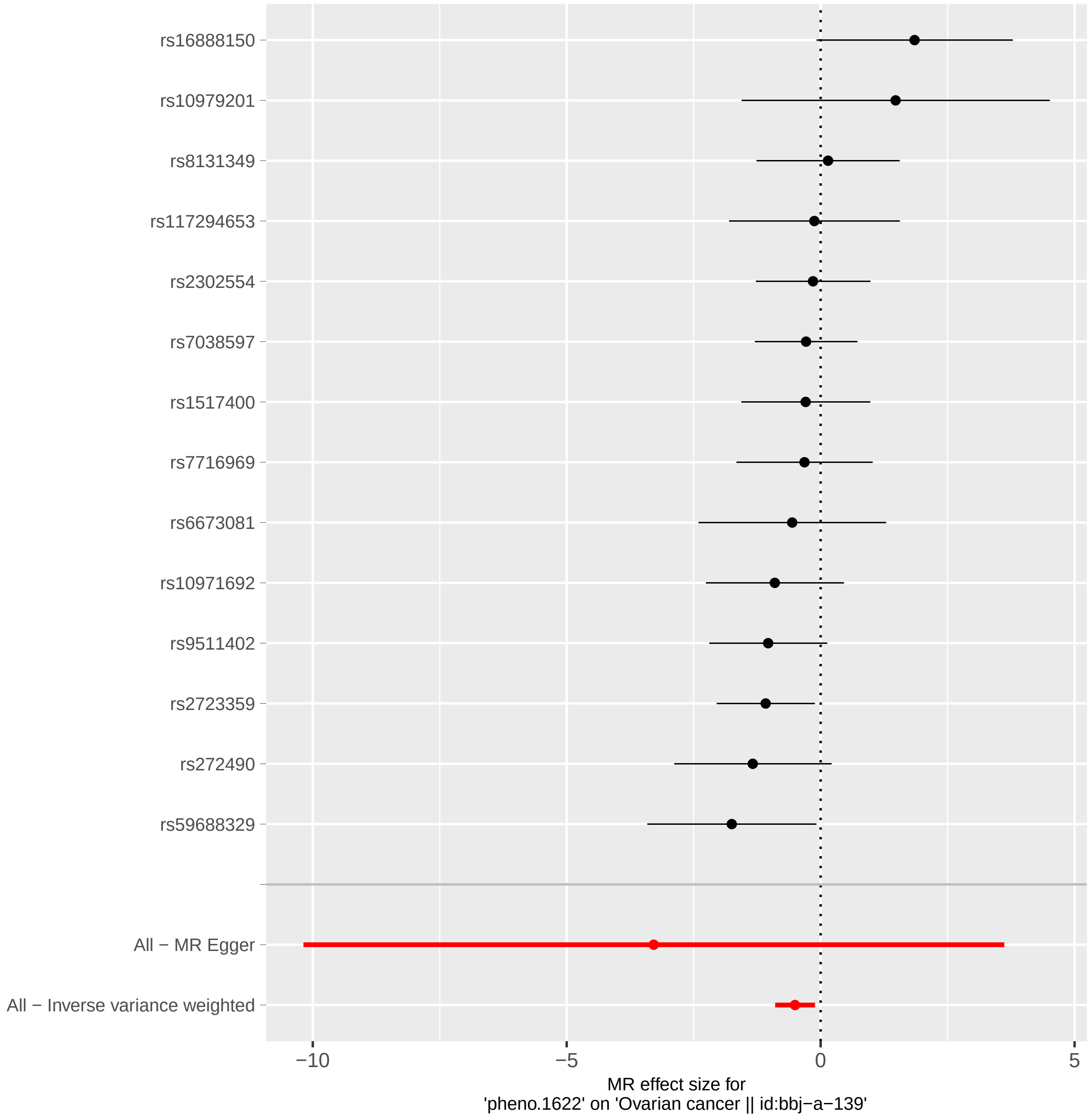
rs16888150
rs10979201
rs8131349
rs117294653
rs2302554
rs7038597
rs1517400
rs7716969
rs6673081
rs10971692
rs9511402
rs2723359
rs272490
rs59688329
All − MR Egger
All − Inverse variance weighted
−10
−5
0
5
MR effect size for
'pheno.1622' on 'Ovarian cancer || id:bbj−a−139'
